# Modeling Ischemia-Reperfusion Injury in Stroke Using the BBB Chip

**DOI:** 10.1021/acsomega.5c06071

**Published:** 2025-09-26

**Authors:** Yunsong Wu, Min Zhang, Peng Wang, Haitao Liu, Xu Zhang, Jianhua Qin

**Affiliations:** † Division of Biotechnology, 58279 Dalian Institute of Chemical Physics, Chinese Academy of Sciences, Dalian 116023, China; ‡ University of Chinese Academy of Sciences, Beijing 101408, China; § University of Science and Technology of China, Hefei 230026, China; ∥ Suzhou Institute for Advanced Research, University of Science and Technology of China, Suzhou 215123, China

## Abstract

Ischemic stroke is a leading cause of disability worldwide and poses a serious threat to public health, affecting tens of millions of people all over the world per year. The lack of a humanized organ model reflecting the real situation of patients has quite limited the pathogenesis research and therapeutic drug development of ischemic stroke. In this study, we developed an ischemic stroke model based on a high-throughput microfluidic chip device, simulating ischemia-reperfusion injury in an ischemic stroke. In this ischemic stroke model, we observed a series of injury characteristics, including significant blood–brain barrier (BBB) destruction, cell apoptosis, and mitochondrial dysfunction. Transcriptome sequencing analysis showed that the expression of genes involved in autophagy, oxidative stress, angiogenesis, and other related pathways was significantly dysregulated in the ischemic stroke model. Drug screening experiments revealed that acetazolamide (AZA), edaravone (EDA), and fasudil (FAS) could significantly reduce the damage in an ischemic stroke model. Overall, the ischemic stroke model recapitulated the physiological and pathological responses of ischemic stroke, and it was innovative that our model focused on reperfusion injury using microfluidic organ-on-a-chip technology. This ischemic stroke brain model holds promise for advancing the development and testing of therapeutic drugs for ischemic stroke, thus contributing to the evolution of treatment strategies.

## Graphical abstract

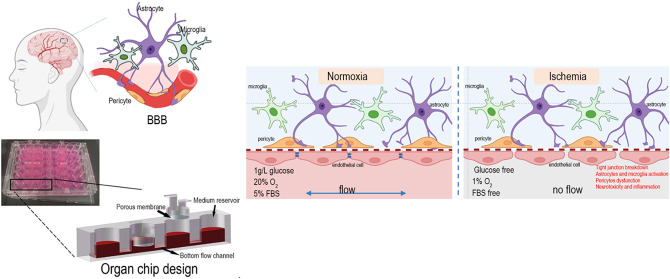

## 1 Introduction

Stroke is recognized as the world’s second leading cause of death and a major contributor to disability, with ischemic stroke accounting for more than 60% of cases.^1^ According to statistics, in 2021, more than 70 million people suffered from ischemic stroke globally.^2^ Ischemic stroke, resulting from the narrowing or occlusion of cerebral arteries, leads to an insufficient blood supply to the brain. This condition causes hypoxia and glucose deficiency in the affected region, resulting in cellular injury and possible disability. The primary goal of ischemic stroke treatment is to restore the blood flow. However, rapid reperfusion of ischemic tissue often leads to reperfusion injury, which is an important contributor to the damage caused by ischemic stroke. The underlying mechanisms involve multiple pathological processes: oxidative stress, infiltration of inflammatory cells, mitochondrial dysfunction, excitotoxicity, apoptosis, and autophagy.^3^ These processes collectively impair the blood–brain barrier (BBB), a vital physiological structure that is crucial for maintaining brain homeostasis. For example, the recovery of oxygen during reperfusion induced an increased production of reactive oxygen species (ROS) and reactive nitrogen species (RNS), which activated the oxidation or nitrosation of downstream proteins that caused further damage to the BBB. Macrophages and neutrophils were stimulated to infiltrate the ischemic region, leading to leakage of the BBB and the onset of cerebral edema.^4^ This highlights the urgent need to investigate BBB dysfunction in the progression of stroke and to develop effective treatment strategies to reduce reperfusion injury. Currently, the primary treatments for stroke include thrombolytic therapy and surgical intervention; however, both of them face a series of challenges, such as a limited window of time and increased intracranial hemorrhage.^5,6^ Moreover, they focus solely on revascularization and reperfusion but ignore neuroprotection against reperfusion injury, leading to a poor prognosis.^7^ Overall, the clinical management of ischemic stroke continues to face challenges due to the lack of effective treatments, largely attributed to the limitations of current in vitro ischemic models in recapitulating reperfusion injury. There is an urgent need to develop effective treatments for ischemic stroke, which is closely related to the establishment of disease models.

Various animal models have been developed to simulate specific stroke pathologies,^8^ such as the vascular occlusion model,^9^ the middle cerebral artery occlusion model,^10^ and the whole blood injection model.^11^ Nonetheless, significant species differences between animals and humans result in notable discrepancies between preclinical and clinical results, ultimately leading to weak predictive capabilities.^12^ Additionally, animal models face challenges, including high costs and operational complexity.^13^ Consequently, there is a critical need to develop in vitro models that closely replicate human physiology and pathology.

A series of in vitro ischemic stroke models have been developed, including BBB models based on transwell devices, dynamic BBB models, organ-on-a-chip-based models, and brain spheroid models. Among them, organ-on-a-chip-based models offer outstanding advantages. Organ-on-a-chip (OOC) technology is a novel approach that creates biomimetic models of human organs on miniature platforms with microfluidic systems to control fluid biology. With the rapid development of OOC over the past ten years, a variety of OOCs have evolved, including models for the lung,^14^ brain,^15^ heart,^16^ liver,^17^ and intestine,^18^ advancing disease modeling research. OOC technology offers extensive application prospects in physiological research, drug development, precision medicine, and other fields. The BBB is a critical tissue interface between the bloodstream and brain parenchyma, and is composed of endothelial cells, pericytes, and glial cells. The structure strictly regulates substance exchange and prevents most harmful agents from entering the brain.^19^ This regulation is essential for maintaining brain microenvironment homeostasis (Figure 1a). BBB-on-a-chip models are promising tools for studying neurological diseases and have been used in research on drug delivery and disease modeling, such as Alzheimer’s disease,^20^ Parkinson’s syndrome,^21^ Huntington’s disease,^22^ traumatic brain injuries,^23^ brain cancer^24^ and brain infection.^25^ However, the application of BBB-on-a-chip in ischemic stroke modeling is still limited.^26^ These models usually lack sufficient structural complexity to simulate the human brain microvascular network, and comprehensive studies of reperfusion injury mechanisms are still lacking. This study developed a physiologically relevant BBB-on-a-chip model for ischemic stroke by incorporating brain microvascular endothelial cells, astrocytes, pericytes, and microglia, which are crucial for BBB formation and function, and further simulate ischemic stroke with oxygen-glucose deprivation stimulation. The oxygen-glucose deprivation (OGD) model is a well-established in vitro model for studying ischemic stroke. Given that the brain is the most oxygen-demanding organ in the human body, its cells rely entirely on a continuous supply of oxygen, glucose, and other nutrients from the bloodstream. During ischemic stroke, cerebral blood flow falls below a critical threshold, causing hypoxia and glucose deprivation in brain cells. These conditions result in a series of cellular damage and stress responses (Figure 1b). Researchers have developed the OGD model by subjecting cells to hypoxia and glucose-deficient conditions to induce the cellular responses observed during ischemic stroke.^27^ This model is advantageous due to its rapid implementation, simplicity, and cost-effectiveness, and this method can also match organ-on-a-chip technology. It has been reported that the BBB-on-a-chip system was used to simulate ischemic stroke using a 3-fold approach, which combined chemical hypoxia, hypoglycemia, and disrupted perfusion, and a significant reduction in barrier function was observed.^28^ However, there is a lack of in vitro models for simulating reperfusion injury.

**Figure 1:**
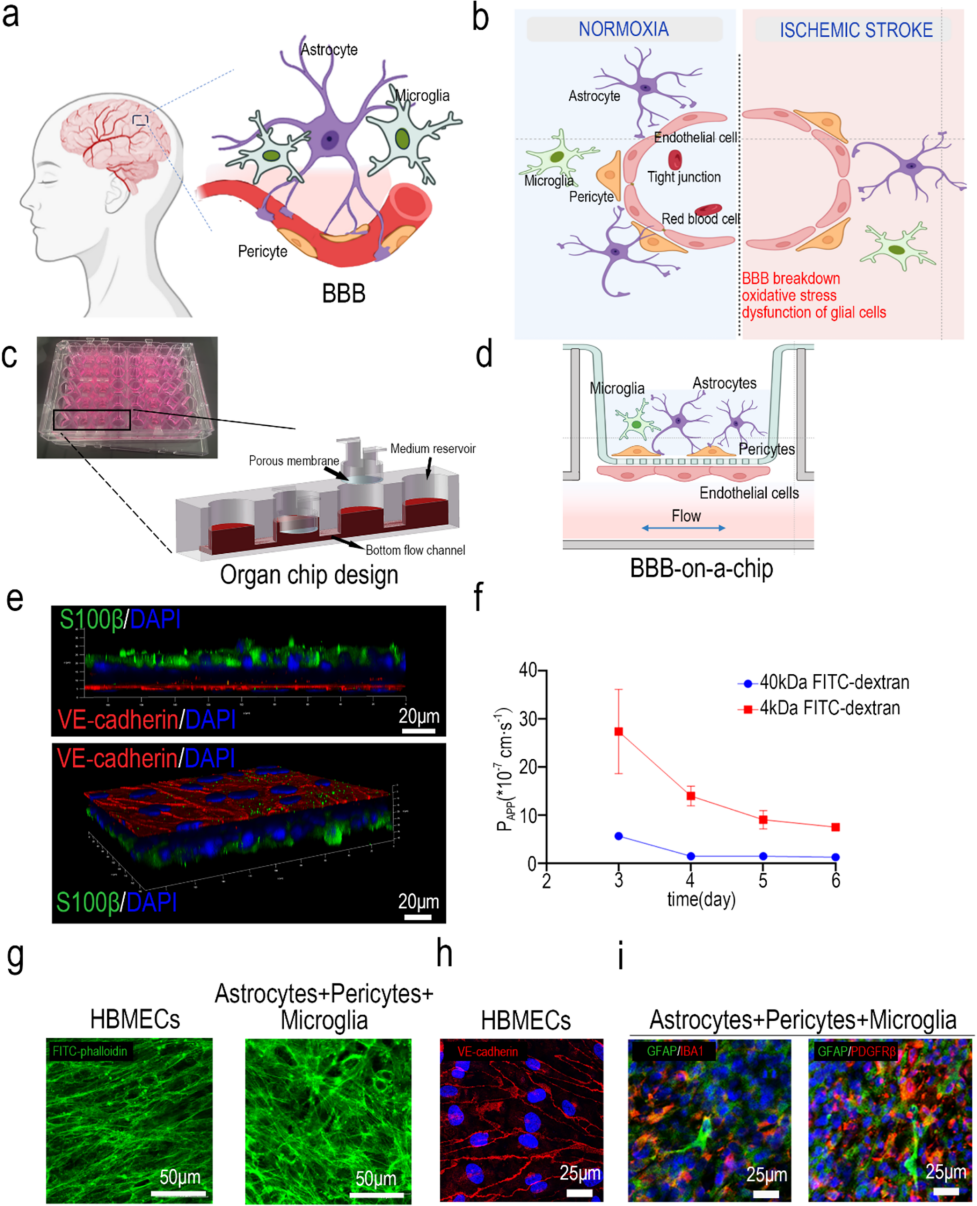
Construction and functional testing of BBB-on-a-chip. (a) Diagram of the BBB. (b) BBB diagram of the normoxia state and ischemic stroke state. (c) Photograph of BBB-on-a-chip and device design. (d) Diagram of BBB-on-a-chip under normoxia and dynamic culture. (e) Side views of the 3D confocal image of the BBB interface on the BBB chip device identified by VE-cadherin in HBMECs and S100β in astrocytes, and 3D confocal image of the BBB chip interface identified by VE-cadherin in HBMECs and S100β in astrocytes. (f) Continuous monitoring of BBB permeability with 4 and 40 kDa FITC-dextran (*n* = 4). (g) Confocal images of the cytoskeleton of cells on both sides of the BBB chips were identified with FITC-phalloidin. (h, i) Representative confocal images of HBMECs immunostained with VE-cadherin, astrocytes immunostained with GFAP, microglia immunostained with IBA1, and pericytes immunostained with PDGDFRβ.

In this study, we established a BBB-on-a-chip system that allows modeling of ischemia-reperfusion injury in ischemic stroke. The model exhibited serious ischemia-reperfusion injury, including a significant increase of apoptosis, breakdown of the endothelial barrier, and oxidative stress. Further research revealed that three drugs were helpful in improving the barrier function and alleviating ischemia-reperfusion injury by inhibiting apoptosis, suppressing oxidative stress, and inhibiting the excessive upregulation of autophagy.

## 2 Results

### 2.1 Construction of a BBB-on-a-Chip System

Our research group developed a high-throughput plug-in chip system to construct a BBB-on-a-chip model, which allows up to 24 OOC units to be cultured simultaneously on a single orifice plate (Figure 1c). The BBB-on-a-chip is composed of two distinct components: the vessel side and the brain side, separated by a porous poly­(ethylene terephthalate) (PET) membrane. The vessel side is located below the porous membrane, and human microvascular endothelial cells (HBMECs) are inoculated on the lower surface of the membrane. The brain side is located above the porous membrane, and the upper surface is seeded with astrocytes, pericytes, and microglia (Figure 1d). Three days after the establishment of the multicellular interaction system, BBB chips are placed on a swing bed for dynamic culture over a period of 3 days. The medium flow on the vessel side of the BBB chips induces fluid stimulation and applies fluid shear stress to HBMECs, which is reported to facilitate the maturation and functionality of the BBB. After 3 days of dynamic culture, confocal microscopic images revealed a dense and continuous expression of adhesion connexin VE-cadherin on HBMECs (Figure 1h), and the cell markers GFAP, PDGFRβ, and IBA1 were expressed in astrocytes, pericytes, and microglia, respectively (Figure 1i). Three-dimensional (3D) reconstruction of immunofluorescence staining images showed the BBB constructed on the chip, which was identified by the continuous VE-cadherin expression in HBMECs on the vessel side and GFAP expression in astrocytes on the brain side (Figure 1e). Examination revealed a decrease of BBB permeability using 4 and 40 kDa FITC-dextran from the vessel side to the brain side every day during the BBB chip culture process, showing the gradual maturation of the barrier function (Figure 1f). Confocal images of the cytoskeleton labeled with FITC-phalloidin indicated the significant difference between the cytoskeleton on the vessel side and the brain side (Figure 1g).

### 2.2 Modeling Ischemia and Ischemia-Reperfusion Injuries on BBB-on-a-Chip

To mimic the characteristics of ischemic stroke, we established ischemia and ischemia-reperfusion models based on our aforementioned BBB-on-a-chip platform. Initially, the culture medium on both sides of the mature BBB chip was replaced with serum-free and glucose-free medium, and the chip was placed in an anoxic incubator with 1% O_2_ and 5% w/v CO_2_ to simulate ischemic conditions. Following this deprivation, oxygen, glucose, and fluid conditions were restored, which is called the OGD/R culture mode, to simulate the reperfusion process typical of an ischemic stroke. The cell viability assessed with the CCK8 assay indicated that ischemia simulation reduced cell viability (Figure 2a). After 4–8 h of OGD, cell viability on both sides of the chip remained above 65% compared with that of the control group. However, a significant decrease in cell viability was observed after 8 h of reperfusion simulation, with the relative viability in the OGD8/R8 h group dropping below 45%, indicative of reperfusion injury in the OGD/R group. Subsequent experiments were performed to model ischemic brain and ischemia-reperfusion brain under the conditions of the OGD8 h and OGD8/R8 h groups, respectively (Figure 2 b). TMRE, a mitochondrial dye, revealed a substantial decrease in the mitochondrial membrane potential in both ischemic brain and ischemia-reperfusion brain models, suggesting cell injury and mitochondrial dysfunction postischemia and reperfusion simulation (Figure 2g,h). Confocal images of the cytoskeleton in HBMECs demonstrated significant damage to the directional fibrous reticular cytoskeleton in both the ischemic brain model and ischemia-reperfusion brain model. Confocal images of TUNEL staining further identified a higher proportion of apoptotic cells in the ischemia-reperfusion brain model compared to both the control and the ischemic brain models, with statistical analysis showing that apoptosis in the ischemia-reperfusion brain model was twice that in the ischemic brain model (Figure 2e,f). Permeability examination revealed a significant increase of BBB chip permeability in the ischemic brain model and ischemia-reperfusion brain model using 4 kDa FITC-dextran from the vessel side to the brain side (Figure 2d). Confocal images reveal a significant downregulation of VE-cadherin expression in HBMECs in the OGD and OGD/R groups, which corroborates the breakdown of the tight junction (Figure 2i). RNA extracted from the vascular side of the chip was analyzed using rt-qPCR to assess the expression of endothelial-related functional genes. Results showed the upregulation of the tight junction protein genes ZO-1 and occludin, adhesion junction protein gene VE-cadherin, and adhesion molecule gene PECAM1 (Figure 2j), possibly reflecting endothelial activation and compensatory mechanisms for barrier breakdown due to reduced tight junctions. The expression of mRNA of the chemokines MCP-1 and cell markers S100β, CD68, and PDGFRβ showed significant changes (Figure S1), which indicated the upregulation of the activation of astrocytes and microglia in the ischemia-reperfusion brain model. Subsequent transmission electron microscopy (TEM) analysis revealed ultrastructural alterations in the BBB models. TEM micrographs (Figure 2k) demonstrated prominent accumulation of autophagosomes in both the ischemic brain model and ischemia-reperfusion model. In contrast, TEM images of normal BBB controls showed no detectable autophagosomal structures. These observations indicate a significant upregulation of autophagic activity in the BBB following OGD and OGD/R treatments. All these findings revealed the significant ischemia-reperfusion injury observed in our ischemia-reperfusion brain model. Further, our research will focus on testing ischemic stroke treatments using this ischemia-reperfusion brain model.

**Figure 2:**
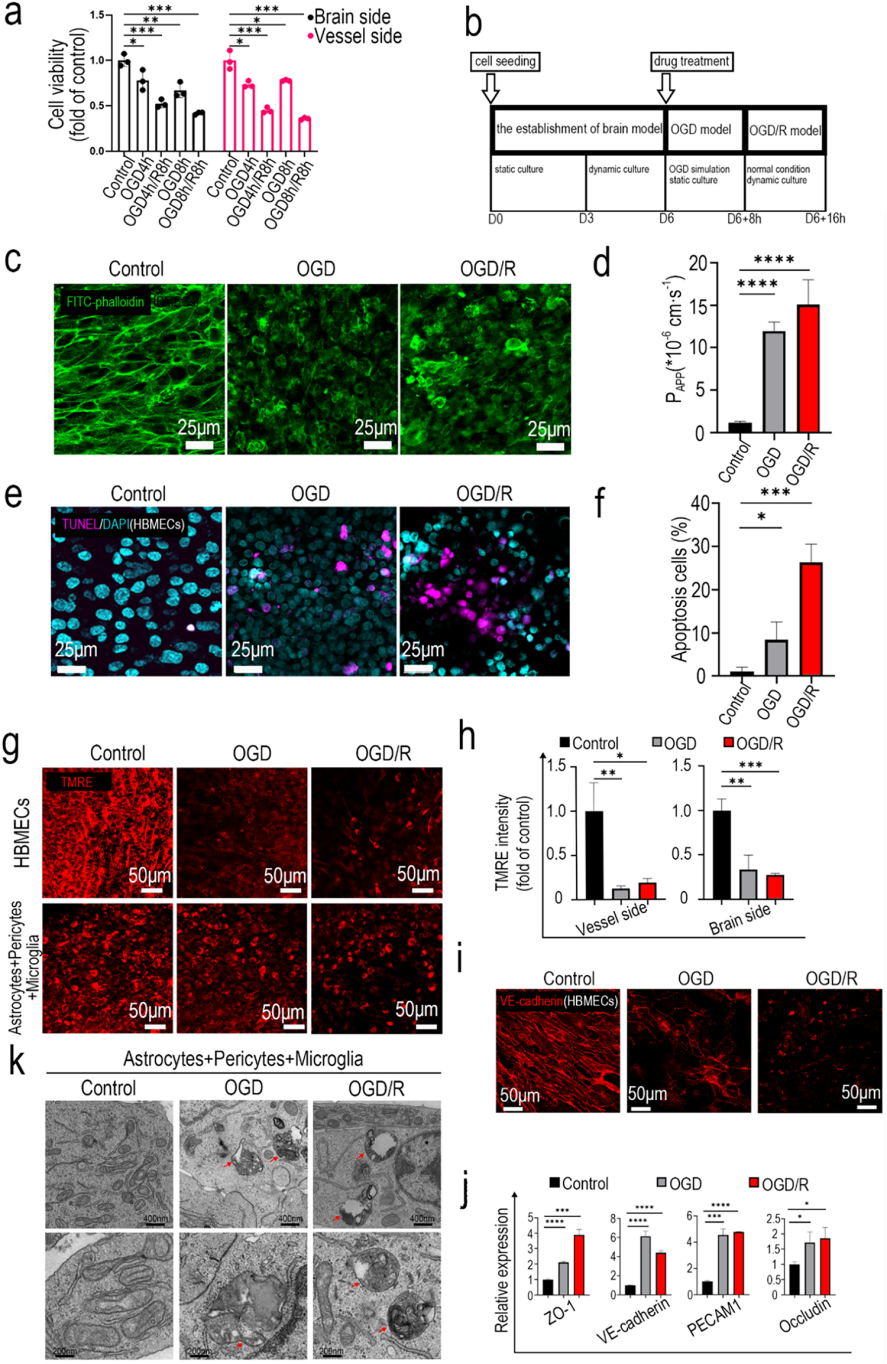
Modeling ischemic simulation or ischemic-reperfusion simulation on a BBB-on-a-chip. (a) CCK8 assay to evaluate OGD-induced and OGD/R-induced cell viability reduction at different injury times. (b) Protocols of OGD and OGD/R models. (c) Confocal images of the cytoskeleton of HBMECs labeled with FITC-phalloidin. (d) BBB permeability analysis with 4 kDa FITC-dextran. (e) Confocal images of apoptotic cells stained with TUNEL assay. (f) Quantitative analysis of apoptotic cells labeled with TUNEL assay. (g) Mitochondrial membrane potential of cells on both sides of the BBB chips stained with TMRE fluorescence. (h) Quantitative analysis of the mitochondrial membrane potential with TMRE fluorescence intensity. (i) Immunofluorescence staining for VE-cadherin in BBB chips of the control group, the OGD group, and the OGD/R group. (j) mRNA levels of ZO-1, VE-cadherin, PECAM1, and occludin were determined by real-time PCR. (k) TEM images of ultrastructural changes following OGD and OGD/R treatments. Red arrows indicate autophagosomes.

### 2.3 Transcriptome Analysis of Cells in Ischemic Stroke Models

Aiming to explore the molecular mechanisms underlying ischemia-reperfusion injury in an ischemia-reperfusion brain model, we analyzed the transcriptome differences between brain and vessel side cells. Briefly, control chips and the ischemia-reperfusion brain models were constructed as previously mentioned, and brain side cells and vessel side cells of each chip were collected and analyzed by RNA-seq. 3194 DEGs were identified on the brain side cells following OGD/R treatment, among which 1937 genes were upregulated and 1257 genes were downregulated (Figure 3a). Then, 3254 DEGs were identified on the vessel side cells, among which 1107 genes were upregulated and 2147 genes were downregulated (Figure 3b). Venn diagrams showed that there were 1386 genes shared between brain side cells and vessel side cells, which revealed that the OGD/R treatment could affect both the side cells in a common manner (Figure 3c). This suggests that there might be some common ischemia-reperfusion injury regulation pathways in cells on both sides of the ischemia-reperfusion brain model.

**Figure 3:**
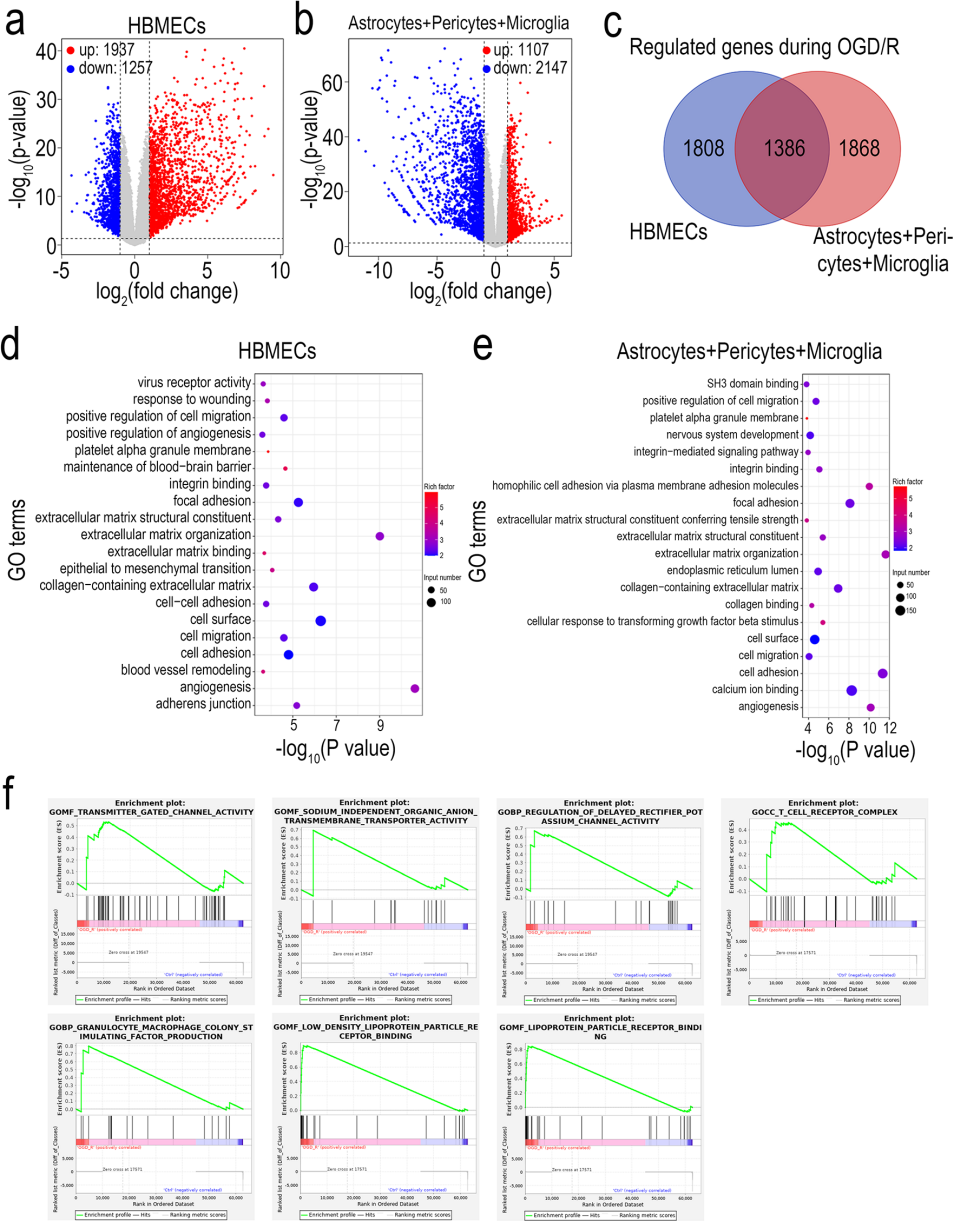
RNA-seq analysis of brain and vessel side cells following OGD/R treatment on the BBB chip. (a, b). Volcano plots showing the regulated genes in brain side cells (a) or vessel side cells (b) following OGD/R treatment on the BBB chip. All DEGs with |logfold-change| > 1 and *p* < 0.05 are marked in color. *p*-Values were calculated using a two-sided, unpaired Student’s *t-*test with equal variance assumed. (c) Venn diagrams depicting the shared or unique DEGs between the brain side and vessel side cells. (d) Gene ontology analysis of DEGs in brain side cells. (e) Gene ontology analysis of DEGs in vessel side cells. In the dot plots of GO analysis, the color of the dots represents the rich factor, and the size of the dots represents the input number of each GO term. (f) GSEA enrichment analysis of OGD/R chips based on the “GO term” reference gene sets.

Next, we conducted gene ontology (GO) enrichment pathway analysis on DEGs to identify the regulated biological processes during the OGD/R treatment (Figure 3d,e). The results showed that OGD/R treatment induced pathways related to integrins, including integrin-binding and integrin-mediated signaling pathways. It was reported that integrin levels increased in ischemic stroke, which is associated with increased ischemia-reperfusion injury.^29^ Compared to nontreated chips, the biological process related to the maintenance of the blood–brain barrier was regulated on the brain side cells in OGD/R chips, and we also found that some modulated genes were enriched in angiogenesis, cell migration, and extracellular matrix-related GO terms in both brain side and vessel side cells, indicating that homeostasis maintenance was significantly affected following OGD/R treatment. In addition, the biological pathways associated with autophagy, apoptosis, and inflammatory responses were also modulated in OGD/R chips. As we know, ischemia-reperfusion injury involves many biological processes, such as apoptosis increase and neuroinflammation (Figures S2 and S3). It has been widely reported that the activation of autophagy during ischemic stroke can cause cell death and also protect the injured site.^30^

To further understand the potential regulatory mechanisms involved in the OGD/R model, GSEA was performed based on the “GO term” reference gene sets. In endothelial cells on the vascular side of the ischemia-reperfusion brain model, we observed multiple gene sets enriched in ion channel-related pathways, including “transmitter-gated channel activity”, “regulation of delayed rectifier potassium channel activity”, and “sodium-independent organic anion transmembrane transporter activity”. Studies have found that the overactivation of potassium-ion channels triggers a fatal cascade reaction, leading to severe neuronal damage,^31,32^ and inhibits the activation of potassium channels in endothelial cells to help alleviate the damage of ischemic stroke.^33^ In the glial cells and pericytes on the brain side of the ischemia-reperfusion brain model, we found that gene sets were enriched in immune-related pathways, including “t-cell receptor complex” and “granulocyte macrophage colony-stimulating factor production”, indicating that the immune response may be enhanced after OGD/R treatment, which leads to increased damage. In addition, we also observed that gene sets were enriched in lipoprotein-related pathways, including “low-density lipoprotein particle receptor binding” and “lipoprotein particle binding”, in glial cells and pericytes on the brain side of the ischemia-reperfusion brain model receptor binding”. The increase of lipoprotein particles, including low-density lipoprotein, is an important factor leading to atherosclerosis,^34^ which may lead to myocardial infarction and stroke,^35^ and targeting lipoproteins can help in treatment.^36^

### 2.4 Drug Testing on the Ischemic Stroke Model

Since one of the main aims of constructing in vitro models of ischemic stroke is to develop a new treatment method, we used our model to verify the efficacy of drugs related to ischemic stroke. Preliminary drug screening was performed at concentrations of 10–20 μM using ten potential therapeutic drugs for ischemic stroke: acetazolamide (AZA), edaravone (EDA), fasudil (FAS), Temozolomide, glibenclamide, imatinib, prednisolone, citicoline, abrocitinib, and ruxolitinib. These agents operate through distinct mechanisms. Among these, some agents have been employed in both clinical trials and preclinical studies for ischemic stroke treatment,^33,37–42^ while others have been applied to ameliorate pathological injuries associated with ischemic stroke.^43^ The main mechanisms of action of these drugs are shown in Table S1. Before exposure to the drugs, the BBB chip was allowed to develop for 3 days, and the cell activity on the BBB chip was compared after 24 h of drug treatment (Figure S4). Among the ten drugs, AZA, EDA, FAS, and Temozolomide treatment did not cause obvious damage to cell activity in BBB chips. The effects of the above drugs on the barrier permeability of the ischemia-reperfusion brain model were further tested (Figure S5). The results showed that Temozolomide had no obvious protective effect on barrier permeability. Therefore, the three drugs, AZA, EDA, and FAS, were initially screened for further study. The action concentrations of the three drugs were adjusted by the preliminary experiment. Examination revealed that the increase of BBB permeability in the ischemia-reperfusion brain model showed notable recovery after treatment with AZA and FAS, which suggests that AZA and FAS would partially restore the barrier function compromised by ischemia-reperfusion injury (Figure 4a). Confocal images show the maintenance of VE-cadherin with EDA treatment, indicating the contribution to the preservation of the tight junction (Figure 4b). Cell viability detection using the LDH assay revealed that three drug treatments could reduce the increase of LDH release in the ischemia-reperfusion brain model (Figure 4g), indicating that these drug treatments contribute to the maintenance of cell viability. Confocal images of apoptotic cells labeled with TUNEL showed that the proportion of apoptotic cells was increased to more than 30%; however, all three drug treatments led to a reduction in this proportion, with EDA and FAS treatments leading to a proportion of less than 10%. (Figure 4c). Furthermore, quantitative analysis of apoptotic cells proved that all three drugs could partially prevent apoptosis in an ischemia-reperfusion brain model, with EDA and FAS treatments being the most effective (Figure 4d). Otherwise, qPCR analysis revealed that all three drug treatments significantly inhibited the upregulated expression of apoptosis-related gene caspase 9 in the OGD/R chips, with FAS treatment also inhibiting the upregulation of caspase 7 (Figure 4h), which indicated that the three drugs were able to alleviate apoptosis.

**Figure 4:**
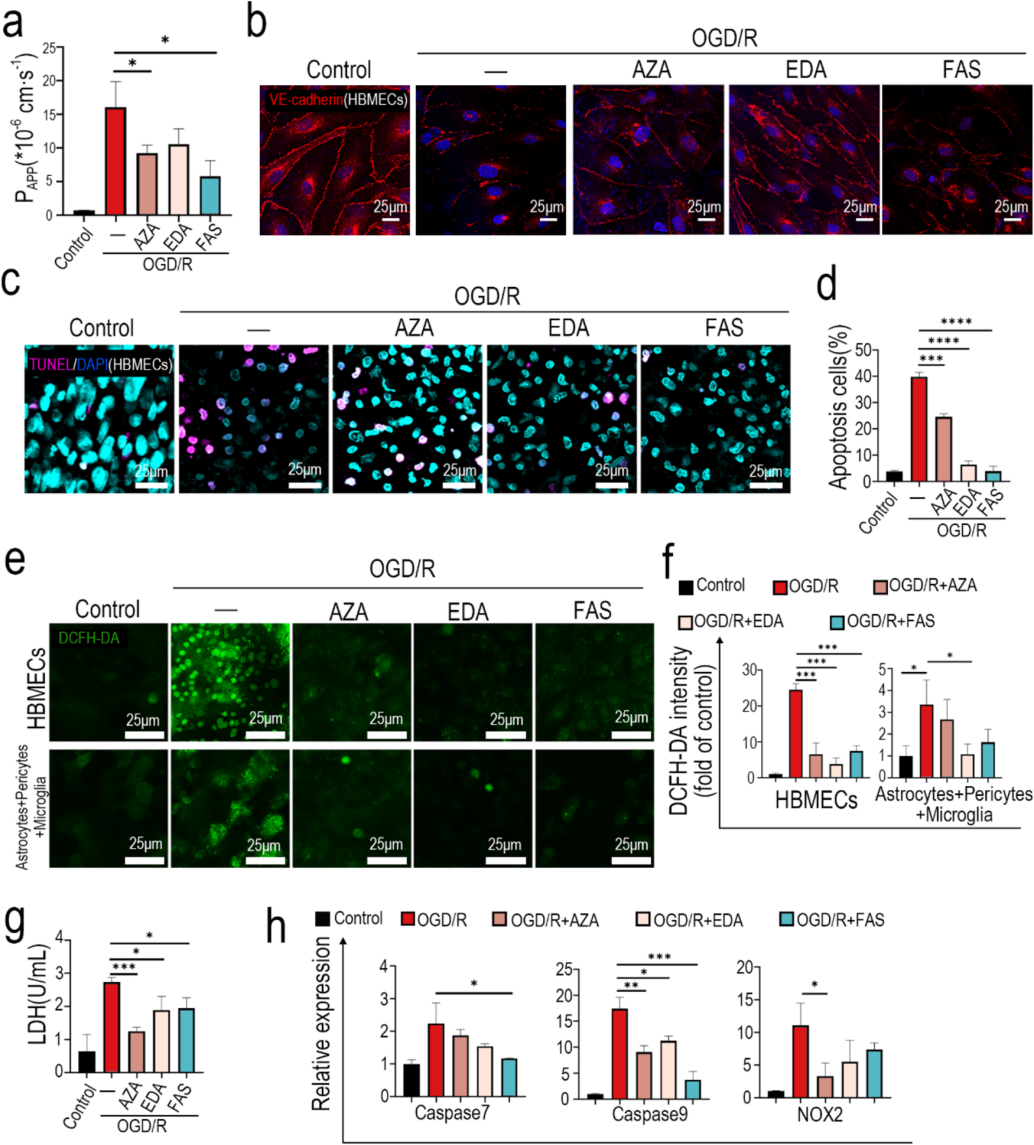
Drug testing on OGD/R chips. (a) BBB permeability analysis with 4 kDa FITC-dextran to evaluate the drug effect on OGD/R chips. (b) Confocal images of HBMECs immunostained with VE-cadherin. (c) Confocal images of apoptotic cells stained with TUNEL assay. (d) Quantitative analysis of apoptotic cells labeled with TUNEL assay. (e) Reactive oxygen species in cells on both sides of the BBB chips were labeled with the DCFH-DA fluorescent probe. (f) Quantitative analysis of ROS levels in BBB chips with DCFH-DA signal intensity. (g) LDH assay to evaluate the protective effect of drugs against the OGD/R-induced decrease of cell viability. (h) mRNA levels of caspase 7, caspase 9, and NOX2 were determined by real-time PCR.

Confocal images showed a significant increase of ROS labeled with DCFH-DA in cells on both the vessel side and brain side of the ischemia-reperfusion brain model. Quantitative analysis of ROS revealed that the upregulation of vessel side cells would be inhibited after drug treatments, and EDA treatment also alleviated the ROS increase on the brain side (Figure 4e,f). Oxidative stress during ischemic stroke is primarily driven by NOX. NOX2 serves as a critical source of ROS in endothelial cells, astrocytes, and microglia.^44^ Following ischemic stroke onset, NOX2 expression is significantly upregulated in both glial cells and endothelial cells.^45^ NOX2-mediated ROS generation exacerbates cerebral injury,^46^ while targeted inhibition of NOX2 significantly attenuates ischemia-reperfusion injury.^47^ QPCR analysis revealed that AZA treatment inhibited the increase in the level of NOX2 expression following OGD/R treatment (Figure 4h).

Further, we hope to explore more pathways and mechanisms by which drug treatments protect the oxidized-state OGD/R chips. IL-1β is an important pro-inflammatory factor, which plays an important role in the aggravation of brain injury caused by ischemic stroke.^44^ ELISA detection of IL-1β levels in the microarray culture medium of each group showed that three drug treatments could inhibit the increase of the IL-1β level in an ischemia-reperfusion brain model (Figure 5a), which showed a reduction in inflammation response. Expression analysis showed that the autophagy-related genes,^48–50^ such as CTSB, CTSD, and VPS41, were significantly upregulated in OGD/R chips, and the upregulation was inhibited following FAS treatment. The expression of VPS41 was further upregulated after AZA treatment (Figure 5b).

**Figure 5:**
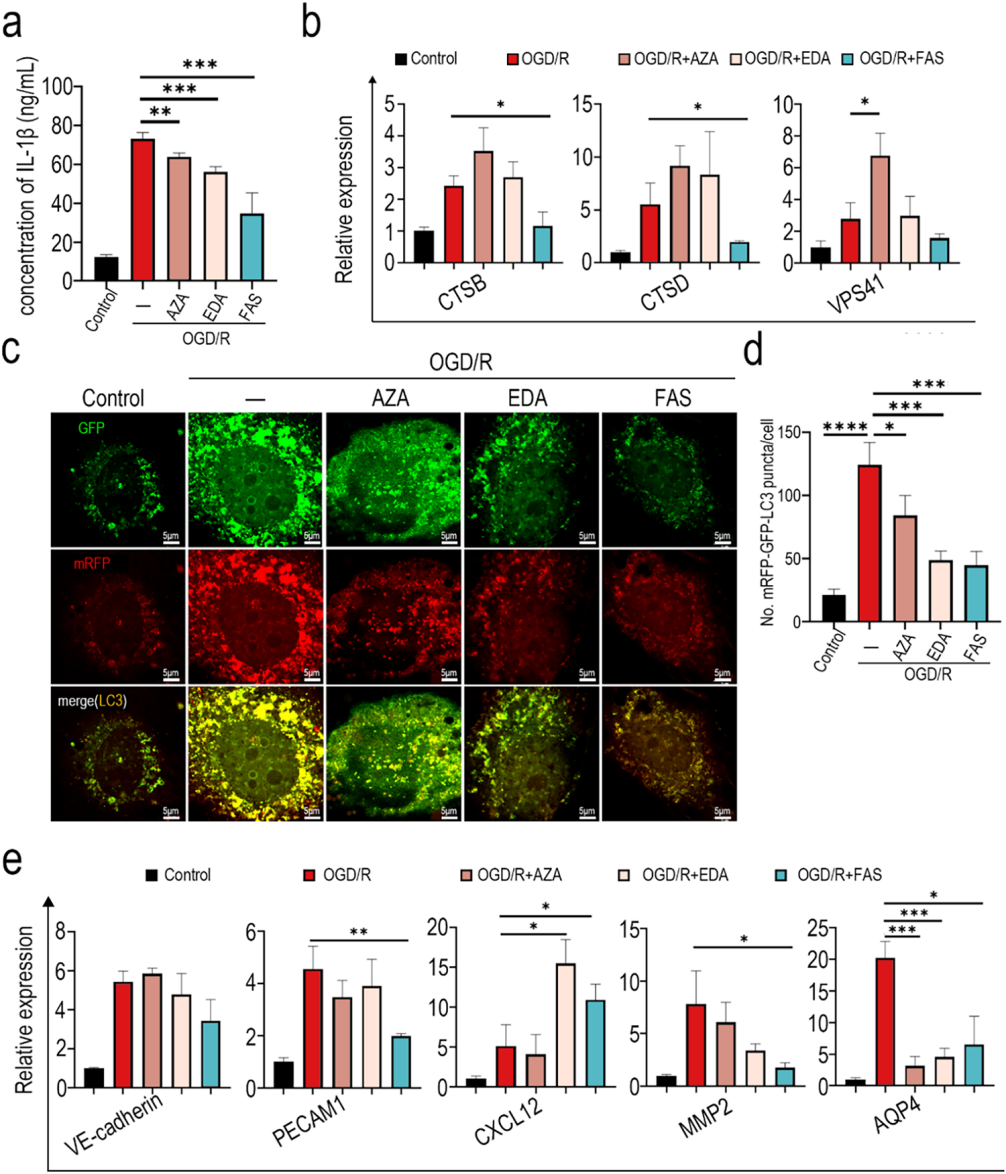
The effect of drug treatment on cell damage caused by OGD/R. (a) ELISA assay to evaluate the levels of the inflammatory factor IL-1β. (b) mRNA levels of autophagy-related genes CTSB, CTSD, and VSP41 were determined by real-time PCR. (c) Confocal images of autophagosomes labeled with HBAD-mRFP-GFP-LC3. (d) Statistical data of autophagosomes identified the mRFP-GFP-LC3 puncta. (e) mRNA levels of VE-cadherin, PECAM1, CXCL12, MMP2, and AQP4 were determined by real-time PCR.

Furthermore, to evaluate autophagosome formation, we used an adenovirus harboring HBAD-mRFP-GFP-LC3 to identify autophagosomes, which are represented by LC3 puncta labeled with both mRFP and GFP. Confocal images of mRFP-GFP-puncta revealed a substantial increase of autophagosomes in OGD/R chips. Meanwhile, EDA and FAS treatments prominently inhibited the increase of autophagosomes, and EDA and FAS significantly prevented apoptosis upregulation in the ischemia-reperfusion brain model.

Then, we examined the expression of functional genes using qPCR (Figure 5e). It was observed that FAS treatment mitigated the upregulation of VE-cadherin and PECAM1 in endothelial cells in the ischemia-reperfusion brain model, indicating the protection of endothelial cells by FAS. CXCL12, a chemokine upregulated in the ischemic penumbral zone, is upregulated in the ischemia-reperfusion brain model and further increased following EDA and FAS treatments, potentially aiding in the functional maintenance and recovery of the brain postischemia-reperfusion injury.^51^ Upregulation of MMP2 is closely related to BBB disruption in stroke.^52^ The analysis of expression of MMP2 showed a significant mitigation of the upregulation in the ischemia-reperfusion brain model following FAS treatment, which contributed to the maintenance of the BBB. Aquaporin-4 (AQP4) expression upregulation in stroke induces brain edema, which causes serious ischemia-reperfusion injury.^53^ Analysis of expression of AQP4 revealed a significant increase in the ischemia-reperfusion brain model; however, all three drug treatments reduced the upregulation, of which AZA, an AQP4 inhibitor, had the most significant inhibitory effect.

## 3 Discussion

In this study, we developed a tetraculture BBB-on-a-chip model based on a plug-in chip platform. The model consists of a vascular side composed of a single layer of cerebral microvascular endothelial cells and a cerebral side composed of a mixed culture of astrocytes, pericytes, and microglia. The fluid channel of the chip platform is used to introduce bionic fluid stimulation. The barrier penetration experiments using FITC-dextran confirmed that the BBB chip had extremely low permeability. Confocal images of endothelial TJP confirmed the formation of a tight endothelial barrier, and confocal images of cell markers showed the establishment of a coculture system on the brain side. Three-dimensional confocal images revealed that the cells on the brain side and the cells on the vessel side were located on both sides of the porous membrane, indicating that the BBB system with ideal cell distribution was constructed. The results show that a BBB chip model with bionic BBB barrier characteristics is established.

BBB-on-a-chip has the advantages of high correlation with human physiology, cost-friendliness, and easy operation, and is suitable for disease modeling research. However, the effect of the BBB chip-based ischemic stroke model is still limited, partly because reperfusion injury is ignored due to the chip structure and other factors. For example, although the BBB chip-based ischemic stroke model reported by Wevers et al. simulated the impairment of cerebral vascular function, it did not explore ischemia-reperfusion injury.^54^ In our ischemia-reperfusion model, we found that the model injury was further aggravated following reperfusion treatment and manifested as reperfusion injury in ischemic stroke. Studies have found that apoptosis and necrosis of cells significantly increase after acute cerebral ischemia, and it is related to many key molecular events of apoptosis, such as ROS increase, Ca^2+^ overload, and excitatory toxicity, and apoptosis leads to nucleus fragmentation, cytoskeleton and nuclear protein degradation, etc.^55,56^ Significant apoptosis and cytoskeletal destruction were also observed in our ischemia-reperfusion brain model, which is consistent with the pathological characteristics of an ischemic stroke. At the same time, RNA-seq analysis results showed that OGD/R treatment induced many biological processes on the BBB chip, among which the regulation of angiogenesis, cell adhesion, barrier maintenance, and endothelial cell medium (ECM) composition may affect the formation and barrier function of the BBB. The enrichment of gene sets related to apoptosis and inflammatory response in cells in the OGD/R chip also reflects the characteristics of ischemia-reperfusion injury. In addition, GSEA of RNA-seq results also found that there were gene sets enriched in ion channel-related, immune-related, and lipoprotein-related pathways in the cells of the ischemia-reperfusion brain model, all of which are related to the onset or aggravation of ischemic stroke.

The endothelial cells that make up the BBB are tightly connected by TJ and AJ to the cytoskeleton of adjacent cells to form a dense barrier, preventing the infiltration of most tissue substances and plasma. MMP2 serves as a critical ligand for low-density lipoprotein receptor-related protein 1 (LRP1). It has been reported that LRP1 regulates multiple tight junction proteins and induces BBB disruption after cerebral ischemia.^57,58^ In clinical studies, MMP2 levels were found to be significantly elevated in ischemic stroke patients compared with those in the control group.^59^ Studies have demonstrated that inhibiting MMP2 activity can substantially mitigate cerebral ischemic injury.^52^ Some studies have also found that there is a BBB destruction pathway independent of MMP in ischemia-reperfusion.^60^ After ischemia-reperfusion, the tension generated by actin polymerization to form stress fibers leads to cytoskeletal rearrangement. The transfer of contractile tension to cell connections anchored to the cytoskeleton leads to the internalization and rearrangement of TJ and AJ, resulting in BBB destruction and fragility.^60,61^ The breakdown of the endothelial connection formed by VE-cadherin was observed in the ischemic brain model and ischemia-reperfusion brain model. The expression analysis of functional genes not only showed that MMP2 was upregulated in the OGD/R chip but also that VE-cadherin, ZO-1, and occludin were upregulated after OGD and OGD/R treatment. Relevant studies have suggested that the upregulation of TJ and AJ expression is a compensatory mechanism of the BBB to counteract cytoskeletal contractility and maintain the barrier.^28^ The results are similar to those of this work, indicating that our chip system can reflect the pathophysiology of endothelial barrier destruction in ischemic stroke.

The development and validation of treatment interventions constitute fundamental applications of pathophysiological disease models. Taking advantage of the high-throughput characteristics of our chip system, we conducted a preliminary screening of ten potential therapeutic drugs, from which three drugs, AZA, EDA, and FAS, with good compatibility and preliminary barrier protection functions, were selected for subsequent research. AQP4 is the most abundant water channel protein in the central nervous system and is crucial for regulating water homeostasis.^62^ Following ischemic stroke, aberrant localization of AQP4 in astrocytes contributes to cerebral edema formation. This pathological process induces severe neurological damage.^63^ In animal models, genetic knockout^64^ or pharmacological inhibition of AQP4 effectively mitigates brain edema and reduces infarct volume.^65,66^ AZA alleviates cerebral edema after ischemic stroke by inhibiting AQP4 and improving collateral circulation.^43^ EDA is a free radical scavenger that can reduce infarct volume and improve nerve function.^67^ Fasudil is a rho-kinase inhibitor approved as a brain protectant for the treatment of cerebral vasospasm in patients with subarachnoid hemorrhage.^68^ Multiple experiments have shown that the three drug treatments were conducive to the maintenance of cell viability and barrier function and inhibited apoptosis. ROS mainly comes from mitochondria, and the mitochondrial electron transport chain (ETC) is interrupted during ischemia, which can produce ROS. Cellular stress during ischemia can induce ETC to be more sensitive to reoxygenation, and reperfusion leads to excess ROS production and accumulation.^69^ This leads to excessive oxidative stress and inflammatory responses, all of which promote neurodegeneration and cell death.^70,71^ By labeling ROS with a DCFH-DA probe, we observed in the ischemia-reperfusion brain model that three drug treatments could partially inhibit the excessive production of ROS caused by OGD/R, revealing that the selected drug treatments can reduce oxidative stress and achieve therapeutic effects.

The role of autophagy in the pathological process of ischemia-reperfusion injury is often described as a double-edged sword. On the one hand, autophagy helps to inhibit inflammatory response,^30,72^ alleviate apoptosis and oxidative stress,^73,74^ maintain and regulate membrane homeostasis and lysosome size.^75^ On the other hand, autophagy is significantly activated in ischemia-reperfusion, leading to autophagy-dependent cell death (ADCD) and aggravating ischemic injury.^76^ It has been reported that ischemic stimulation can significantly induce autophagy of astrocytes,^77^ microglia,^78^ and endothelial cells,^79^ leading to serious injury. Studies have shown that inhibition of autophagy can significantly reduce cell death during ischemia.^80,81^ Similarly, we observed a significant upregulation in the number of autophagosomes and the expression of autophagy-related genes on the ischemia-reperfusion brain model, indicating that OGD/R treatment significantly induced autophagy, and we found that EDA and FAS treatment could inhibit the excessive upregulation of autophagy. Many studies on the regulation of autophagy by EDA and FAS have shown that EDA can inhibit the activation and apoptosis of glial autophagy in the ischemia-reperfusion process,^82–84^ and it has also been reported that EDA can promote angiogenesis, inhibit oxidative stress, and apoptosis by activating autophagy.^85^ The mechanism of FAS alleviating ischemia-reperfusion injury by activating cardiomyocyte autophagy has also been reported.^86,87^ High expression of apoptosis in our model may correspond to overexpression of autophagy. Transcriptome analysis of the ischemia-reperfusion brain model also showed that the expression of genes enriched in autophagy-related biological processes was significantly upregulated in cells on the brain side in the ischemia-reperfusion brain model. However, EDA and FAS treatments can alleviate ischemia-reperfusion brain model damage, and the inhibitory effect on the upregulation of autophagy suggests that EDA and FAS can inhibit overactivated autophagy and contribute to the maintenance of BBB activity and function.

Ischemic stroke remains a major health threat globally, necessitating extensive research into its pathogenesis and the development of effective treatments. Our study demonstrates that the stroke-on-chip mode offers significant advantages in terms of efficiency, cost-effectiveness, and physiological relevance. Researchers can interconnect different types of organ chips to investigate broader pathophysiological processes involving multiple organs, which is beneficial for advancing stroke research. Looking ahead, it is crucial to develop a wider variety of stroke models to address clinical needs, such as models for aging populations and age-related diabetic patients. This will enhance the clinical value of extracorporeal ischemic stroke models.

## 4 Conclusions

This study describes a tetraculture BBB-on-a-chip model that utilizes a plug-in chip platform to replicate the ischemia and ischemia-reperfusion states associated with the onset of ischemic stroke. The model effectively demonstrated significant cell damage following ischemia, with further exacerbation of damage upon reperfusion, which is characterized by barrier destruction, apoptosis, oxidative stress, inflammatory responses, and overactivation. These observations align with the pathological findings in patients suffering from ischemic stroke. Additionally, we employed this model to evaluate ischemic stroke drugs, identifying multiple pathways through which several potential drugs ameliorate ischemia-reperfusion injury. This work provides a unique platform for studying the pathophysiology of ischemic stroke and developing new therapeutics for this prevalent neurological disease.

## 5 Materials and Methods

### 5.1 Cell Culture

hBMEC cells were cultured in ECM (ScienCell, #1001), and HA cells were cultured in AM (ScienCell, #1801). Pericytes were cultured in PM medium (ScienCell, #1201). HMC3 cells (Procell, #HMC3) were cultured in MEM (Procell, #PM150410) supplemented with 10% fetal bovine serum (FBS) (Gibco, #10099-141) and 1% P/S. All kinds of media were supplemented with 0.2% plasmocin treatment (Invivogen, #ant-mpp)

### 5.2 Immunostaining

Immunostaining was performed as previously described.^88^ Cells were fixed with 4% PFA for 20–30 min and then washed with phosphate-buffered saline (PBS; Meilunbio, #MA0015) three times for 5 min. Next, the sample was incubated with 0.2% Triton X-100 for 10 min. The cells were blocked with 5% normal goat serum for 30 min and incubated with primary antibodies overnight at 4 °C. Then, cells were incubated with secondary antibodies for 1 h at room temperature. After staining with antibodies, the nuclei were stained with DAPI. Images were acquired with an Olympus FV3000 confocal fluorescence microscope system.

### 5.3 Permeability Test with FITC-Dextran

The permeability test of the BBB model was performed as previously described.^88^ In short, 2 mL of ECM containing 25 μg/mL FITC-dextran (40 kDa) was infused into the bottom layer, and BBB chips were placed on a shaker. 1 h later, the medium on both sides of the BBB chip was collected and measured with a plate reader (Infinite 200 PRO; TECAN, #INFINITE M NANO+) at 488 nm (excitation) and 530 nm (emission) for FITC-dextran concentration determination.

The apparent permeability coefficient Papp was calculated according to the following equation

$$P_{\mathrm{app}}=\frac{V_{g}C_{g}}{AC_{\mathrm{en}}t}$$

where *V*_g_ (mL) and *C*_g_ (mol/mL) are the volume and concentration of FITC-dextran in the glial channel, respectively; *A* (cm^2^) is the contact area between the endothelial channel and glial channel; *C*_en_ (mol/mL) is the FITC-dextran concentration in the endothelial channel; and *t* (s) is the diffusion time of FITC-dextran.

### 5.4 OGD and OGD-RE Treatment in the BBB Chip

As mentioned above, oxygen and glucose deprivation (OGD) was used to simulate ischemic stroke. Cells were incubated in glucose- and serum-free DMEM or ECM at 1% O_2_ and 5% CO_2_ in a low-oxygen incubator. Reperfusion (OGD-RE) was simulated by incubating cells in a medium containing usual concentrations of glucose and serum at 21% O_2_ and 5% CO_2_ in an incubator.

### 5.5 RT-qPCR Analyses

One milliliter of TRIzol reagent was infused into each unit of the BBB chip, blowing both sides of the film repeatedly for 2 min. Each group of lysates was collected separately, and total RNA was isolated to construct a cDNA library. RNA concentration and quality were assessed by examining A260/A280 with a plate reader (Infinite 200 PRO; TECAN, #INFINITE M NANO+). A cDNA library was constructed using the PrimeScripTMRT Reagent Kit with gDNA Eraser (TaKaRa, #RR047A) according to the manufacturer’s instructions. Expression of target genes and GAPDH as reference was analyzed by real-time qPCR using TB Green Premix Ex TaqTM II (TaKaRa, #RR820A) according to the manufacturer’s recommendations in a real-time fluorescence quantitative PCR instrument. GAPDH was used as a reference gene.

### 5.6 LDH Assay

Cytotoxicity was assessed using the LDH Cytotoxicity Assay Kit with WST-8 (Beyotime, #C0018S) according to the manufacturer’s recommendations.^89^ Absorbance was measured at 450 nm minus 600 nm with a plate reader (Infinite 200 PRO; TECAN, #INFINITE M NANO+).

### 5.7 Cell Viability Assay Using EthD-1/Calcein AM

Live cells and dead cells were assessed using LIVE/DEADTM Viability/Cytotoxicity Kit (Invitrogen, #2261453) according to the manufacturer’s recommendations.^90^ Live cells were labeled with calcein AM, which was excited by light at a wavelength of 488 nm. Dead cells were labeled with EthD-1, which was excited by light at 594 nm. Images were acquired with an Olympus FV3000 confocal fluorescent microscope system.

### 5.8 Cell Viability Assay Using CCK8

Cell viability was assessed with Enhanced Cell Counting Kit-8 (Beyotime, #C0042).^91^ The mixed culture medium and CCK8 reagent were mixed in a ratio of 9:1, and the mixed medium was added directly to the cells. Cells were cultured at 37 °C for 2 h, the medium was collected, and the absorbance was measured at 480 nm with a plate reader (Infinite 200 PRO; TECAN, #INFINITE M NANO+).

### 5.9 Mitochondrial Membrane Potential Detection with TMRE

TMRE (tetramethylrhodamine ethyl ester) is an orange cationic fluorescent probe that penetrates the cell membrane. TMRE aggregates in normal mitochondria, which have a lot of negative charge inside, and a bright fluorescent signal is visible under a confocal microscope, whereas mitochondrial membrane potential loss that TMRE was released, and the fluorescent signal drops. A TMRE (Beyotime, #C2001S) working solution was prepared according to the manufacturer’s recommendations and was used to then stain the cells on the BBB chip at 37 °C for 30 min.^92^ The fresh medium was replaced after staining, and the images of the TMRE fluorescent signal were acquired with an Olympus FV3000 confocal fluorescent microscope system.

### 5.10 ROS Detection with DCFH-DA

Dichlorodihydrofluorescein diacetate (DCFH-DA) is a nonpolar substance that penetrates the cell membrane. Inside the cell, DCFH is oxidized to a highly fluorescent substance, dichlorodihydrofluorescein (DCF), by intracellular oxidants such as H_2_O_2_. The DCFH-DA reagent was diluted with serum-free cell medium, and the medium in the BBB chip was replaced with it. The BBB chip was incubated at 37 °C for 30 min without light and then washed with fresh serum-free medium.^93^ Images with fluorescent signals were acquired using an Olympus FV3000 confocal fluorescent microscope system.

### 5.11 FITC-Phalloidin Staining

Cells were washed twice with PBS and fixed with 4% PFA for 20–30 min. Next, the cells were treated with 0.2% Triton X-100 for 20 min for permeabilization, and blocked with 5% goat serum for 30 min. Then, cells were incubated with FITC-Phalloidin (GLPBIO, #FITC-Phalloidin) (1:1000) for 1 h to stain the cytoskeleton according to the manufacturer’s recommendations, followed by washing with PBS twice.^94^ The stained cytoskeleton was observed with an Olympus FV3000 confocal fluorescence microscope system, and the images were acquired.

### 5.12 Autophagosomes Labeling with HBAD-mRFP-GFP-LC3 Adenovirus

The formation of autophagosomes is a key indicator for the evaluation of the level of autophagy, and tracing autophagosomes with GFP/RFP-LC3 fusion protein is a common method. After a series of experiments, including the pretest of multiplicity of infection (MOI) of HBAD-mRFP-GFP-LC3 adenovirus (HANBIO, # HBAD-mRFP-GFP-LC3), the infection of cells on BBB chips, 36-h culture of normal BBB chips, ischemic BBB model, and ischemia-reperfusion BBB model, the fixation with PFA, and treatment with sealant on chips, according to the manufacturer’s recommendations, the level of autophagosomes that were labeled with both GFP and mRFPwere evaluated with an Olympus FV3000 confocal fluorescent microscope system, and the images were acquired.^95^

### 5.13 Apoptotic Cells Labeling with TUNEL Assay

The principle of the TdT-mediated dUTP Nick-End Labeling (TUNEL) method is that fluorescein-dUTP is connected to the exposed 3′-OH of broken DNA during apoptosis by TdT, and then the fluorescent signal of apoptotic cells can be observed using fluorescence microscopy. After a series of experiments including washing cells with PBS, fixation with fixation buffer, permeabilization with permeabilization buffer, balance of cells with TdT equilibration buffer, incubation with labeling solution in without light for 60 min, stop solution and washing with PBS, according to manufacturer’s recommendations (Elabscience, #E-CK-A424), the apoptotic cells were observed with an Olympus FV3000 confocal fluorescence microscope system and images were acquired.

### 5.14 RNA-Seq and Analysis

RNA-seq was performed as previously described.^88^ Total RNA was isolated from both sides of the chips, respectively, with TRIzol reagent (Thermo Scientific, cat. #15596026). Subsequent to RNA extraction, genomic DNA was removed using DNase I (NEB, cat. #M0303L) treatment to eliminate potential DNA contamination, and the purity of the extracted RNA was measured using a Nanodrop TM OneC spectrophotometer (Thermo Scientific). The integrity of the RNA was verified using the LabChip GX Touch system (Revvity), and the concentration was determined using a Qubit 3.0 fluorometer with the QubitTM RNA Broad Range Assay kit (Thermo Scientific, cat. #Q10210). Next, total RNA was used as input for RNA sequencing library preparation utilizing the KCTM mRNA Library Prep Kit (Seqhealth Tech. Co., Ltd., Wuhan, China) according to the manufacturer’s instructions. Fragments ranging from 200 to 500 bp were enriched, quantified, and sequenced on a DNBSEQ-T7 platform (MGI) using the PE150 sequencing mode to generate paired-end reads.

Differentially expressed genes and expressed transcripts between groups were identified using the edgeR package (version 3.40.2), with a p-value cutoff of 0.05 and a fold-change cutoff of 2. Gene ontology (GO) analyses for DEGs were implemented using KOBAS software (version: 2.1.1) with a *p*-value cutoff of 0.05 to determine statistically significant enrichment. Gene Set Enrichment Analysis (GSEA) was performed using the GSEA software with the GO and KEGG background data sets from the MSigDB database.

### 5.15 Transmission Electron Microscopy

Fixed BBB chips were treated with 2.5% glutaraldehyde (Electron Microscopy Sciences) at 4 °C overnight. After washing with PBS and fixation with 1% OsO_4_ for 2 hours, the BBB chips were dehydrated using graded ethanol solutions. Next, the chips were embedded in Epon812 resin (SPI) to obtain ultrathin sections. Then, the sections were stained with 2% uranyl acetate for 30 min and with lead citrate for 10 min. Images were acquired using a JEM-1400PLUS electron microscope system.

### 5.16 Determination of IL-1β by ELISA

According to the manufacturer’s recommendations, IL-1β secreted by cells was assessed using the ELISA MAX Deluxe Set Human IL-1β (BioLegend, #437004). The medium was collected from the chips, and IL-1β was assessed using an ELISA Kit. The absorbance was measured at 450 nm and subtracted from the absorbance at 570 nm using a plate reader (Infinite 200 PRO; TECAN, #INFINITE M NANO+).

### 5.17 Fabrication of BBB-on-a-Chip System

The BBB-on-a-chip system in this study was based on a microfluidic chip with a 48-well plate and a sextet transwell-like insertions, which was designed and assembled by our research group. A PET porous film (5 μm aperture and 10 μm thickness) was glued on the bottom of the insertion with plasma desorption mass spectrometry (PDMS) and cropped by a laser cutter. Next, the assembled microfluidic chip was exposed to ultraviolet (UV) light overnight for sterilization.

Before cell culture, the PET porous film was coated with 1% collagen type I at 37 °C for 2 h to facilitate the adhesion of cells on both sides of the film. hBMECs were first seeded on the lower surface of a PET porous film at a density of 5 × 10^6^ cells/mL to construct the endothelial layer. Three hours after hBMECs were seeded, HA cells and pericytes mixed in equal parts were seeded at 2 × 10^6^ cells/mL on the upper layer of the PET porous film. Two hours later, hMC3 cells were seeded at 4 × 10^5^ cells/mL on the upper layer of the PET porous film. The glial cell layer was composed of HA cells, pericytes, and hMC3 cells. Three days later, the BBB-on-a-chip was put on a shaker to mimic the fluid and create physiological shear stress in the BBB.

### 5.18 Statistical Analyses

Data were analyzed using GraphPad Prism 6 software. Differences between the two groups were analyzed using an unpaired Student’s *t-*test. Multiple group comparisons were performed using one-way analysis of variance (ANOVA), followed by Bonferroni post hoc tests. Data are presented as mean ± standard error of the mean (SEM). Significance is indicated by asterisks: *p < 0.05, **p < 0.01, and ***p < 0.001.

## Supplementary Material



## Notes

mRNA levels of MCP-1, S100β, CD68, and PDGFRβ were determined by real-time PCR (Figure S1); gene ontology (GO) analysis of upregulated genes of astrocytes, pericytes, and microglia in the ischemia-reperfusion brain model, and different colors represent different functional classifications (Figure S2); gene ontology (GO) analysis of upregulated genes of HBMECs in ischemia-reperfusion brain model, and different colors represent different functional classifications (Figure S3); CCK8 assay to evaluate cell viability in the BBB chip following different drug treatment (Figure S4); BBB permeability analysis with 4 kDa FITC-dextran to preliminarily evaluate the effect of AZA, EDA, FAS, and Temozolomide on OGD/R chips (Figure S5); and summary of drug compounds and mechanisms of drug action (Table S1) (PDF)

## Abbreviations

ADCD: autophagy-dependent cell death
AJ: adherens junction
AM: astrocyte medium
AQP4: aquaporin-4
AZA: acetazolamide
BBB: blood–brain barrier
CCK8: cell counting kit-8
CD68: cluster of differentiation 68
CTSB: cathepsin B
CTSD: cathepsin D
CXCL12: chemokine (C-X-C motif) ligand 12
DCFH-DA: 2′, 7′-dichlorodihydrofluorescein diacetate
DEG: differentially expressed gene
ECM: endothelial cell medium
EDA: edaravone
ELISA: enzyme-linked immunosorbent assay
ETC: electron transport chain
FAS: fasudil
FBS: fetal bovine serum
FITC: fluorescein isothiocyanate
GFAP: glial fibrillary acidic protein
GFP: green fluorescent protein
GO: gene ontology
GSEA: gene set enrichment analysis
HBAD-mRFP-GFP-LC3: Hanbio adenovirus encoding mRFP-GFP-LC3
HBMEC: human microvascular endothelial cell
IBA1: ionized calcium-binding adapter molecule 1
JAK/STAT: Janus kinase/signal transducer and activator of transcription
KEGG: Kyoto encyclopedia of genes and genomes
LC3: microtubule-associated protein 1*A*/1B-light chain 3
LDH: lactate dehydrogenase
MCP-1: monocyte chemotactic protein 1
MEM: Minimum essential medium
mRFP: monomeric red fluorescent protein
MMP: matrix metallopeptidase
MMP2: matrix metallopeptidase 2
NOX2: NADPH oxidase 2
OGD: oxygen-glucose deprivation
OGD/R: oxygen-glucose deprivation/reperfusion
OOC: organ-on-a-chip
PDGFRβ: platelet-derived growth factor receptor β
PECAM1: platelet endothelial cell adhesion molecule
PET: poly­(ethylene terephthalate)
PM: pericyte medium
qPCR: quantitative real-time polymerase chain reaction
RNS: reactive nitrogen species
ROS: reactive oxygen species
rt-qPCR: reverse transcription quantitative real-time polymerase chain reaction
S100β: S100 calcium-binding protein B
TEM: transmission electron microscope
TJ: tight junction
TMRE: tetramethylrhodamine ethyl ester
TUNEL: terminal deoxynucleotidyl transferase-mediated dUTP Nick-End Labeling
VPS41: vacuolar protein sorting-associated protein 41
