# Supplementary material for: Modeling Ischemia-Reperfusion Injury in Stroke Using the BBB Chip
Source: ACS Omega. 2025 Sep 26;10(39):45680–95. doi: 10.1021/acsomega.5c06071 (PMC12508954; doi:10.1021/acsomega.5c06071)
Supplement: Supplementary file 1 [file ao5c06071_si_001.pdf]

Modeling ischemia-reperfusion injury in stroke using BBB chip

Supplementary Information

Yunsong Wu[1,2], Min Zhang[1], Peng Wang[3,4], Haitao Liu[1], Xu Zhang[1], Jianhua Qin[1,2,3,4]*

[1] Division of Biotechnology, Dalian Institute of Chemical Physics, Chinese Academy of Sciences, Dalian, China.
[2] University of Chinese Academy of Sciences, Beijing, China
[3] University of Science and Technology of China, Hefei, China
[4] Suzhou Institute for Advanced Research, University of Science and Technology of China, Suzhou, China
[*]Correspondence: E-mail: jhqin@dicp.ac.cn; Tel: 86-0411-84379650; Division of Biotechnology, Dalian Institute of Chemical Physics, Chinese Academy of Sciences, 457 Zhongshan Road, Dalian 116023, China.

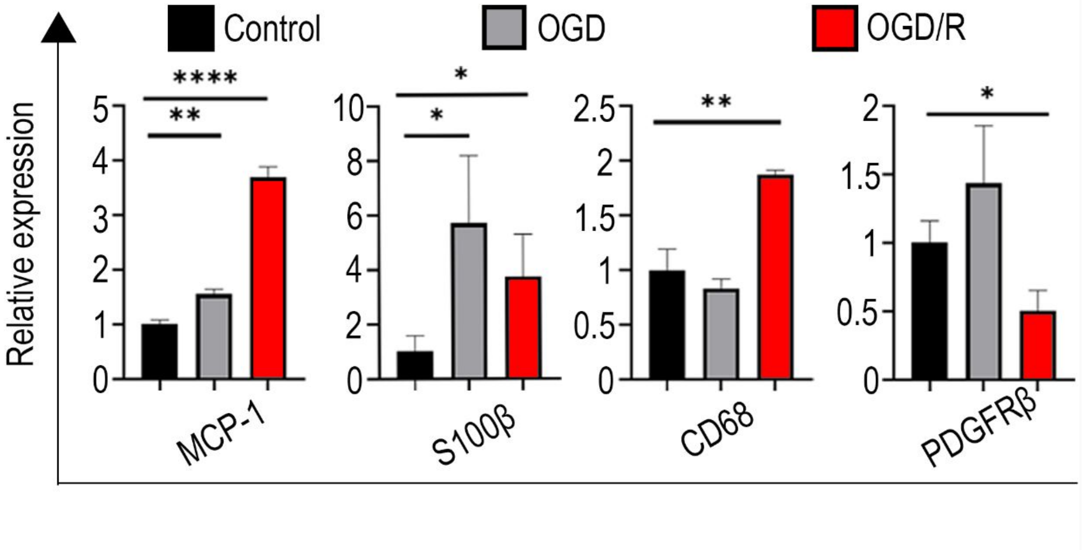

Figure S1. The mRNA levels of MCP-1, S100β, CD68 and PDGFRβ were determined by real-time PCR.

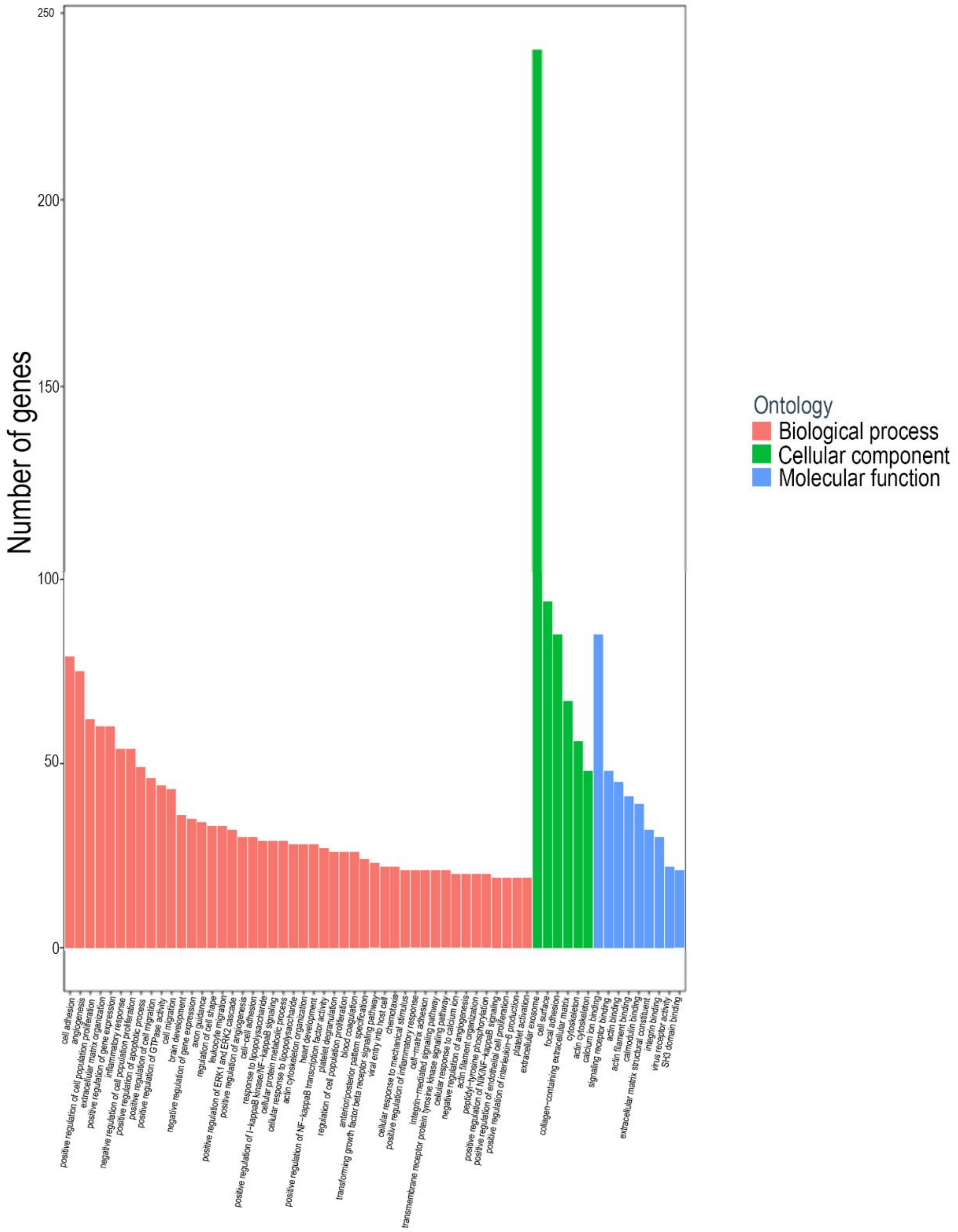

Figure S2. Gene Ontology (GO) analysis of up-regulated genes of astrocytes, pericytes and microglia in ischemia-reperfusion brain model. Different colors represent different functional classification.

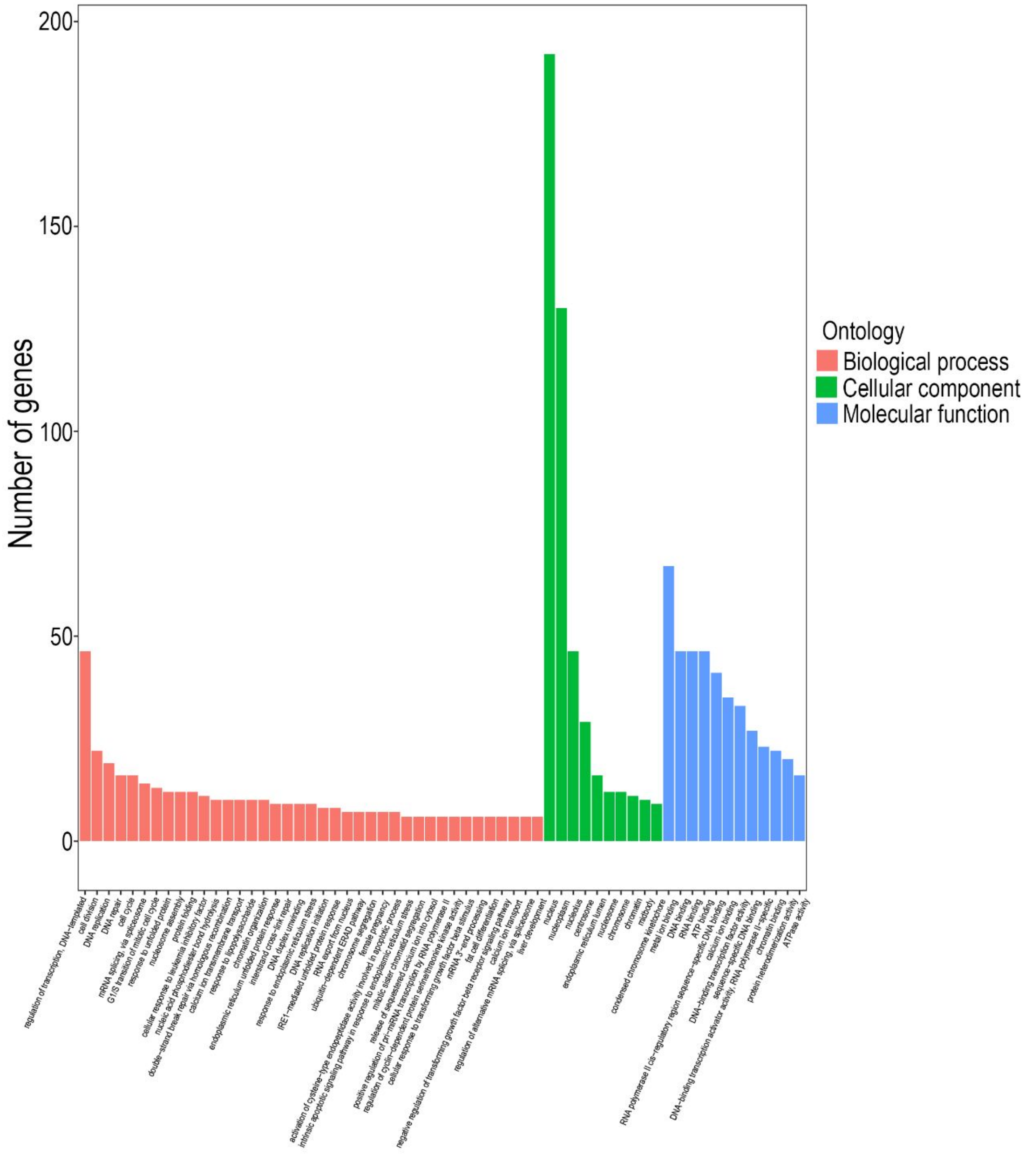

Figure S3. Gene Ontology (GO) analysis of up-regulated genes of HBMECs in ischemia-reperfusion brain model. Different colors represent different functional classification.

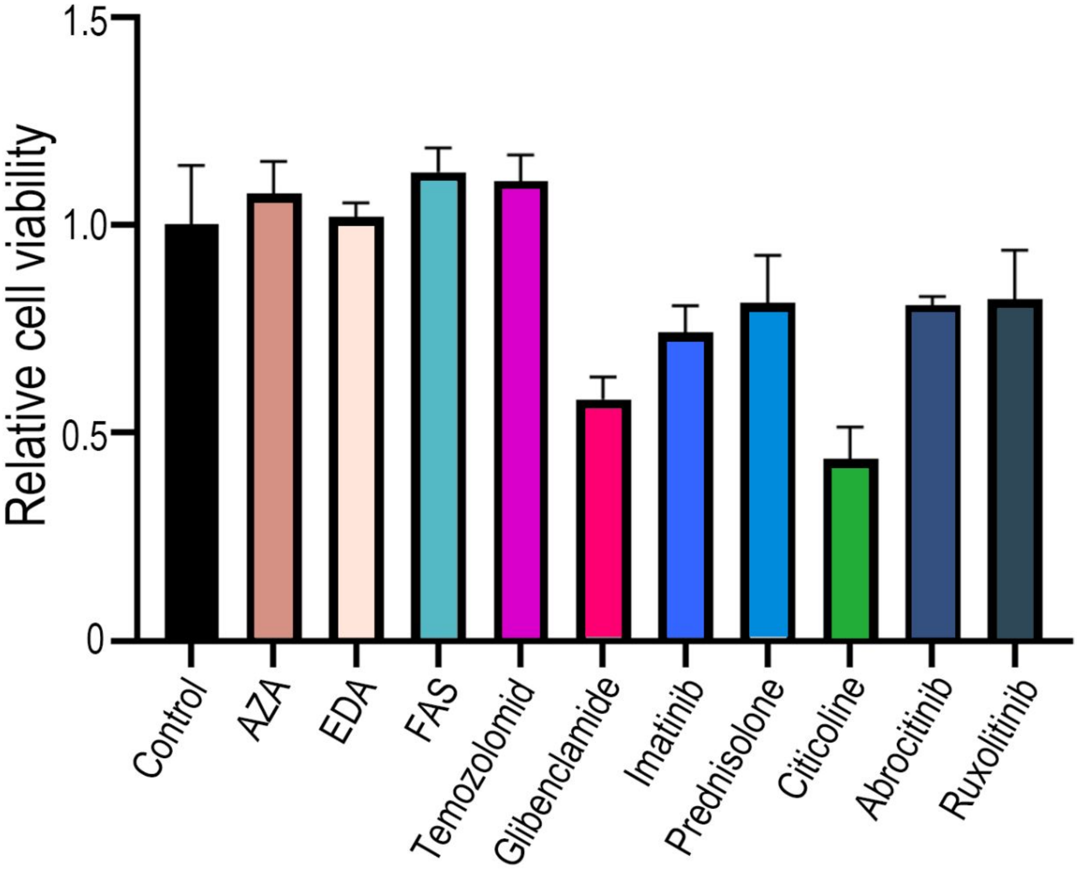

Figure S4. CCK8 assay to evaluate the cell viability in BBB chip following different drugs treatment.

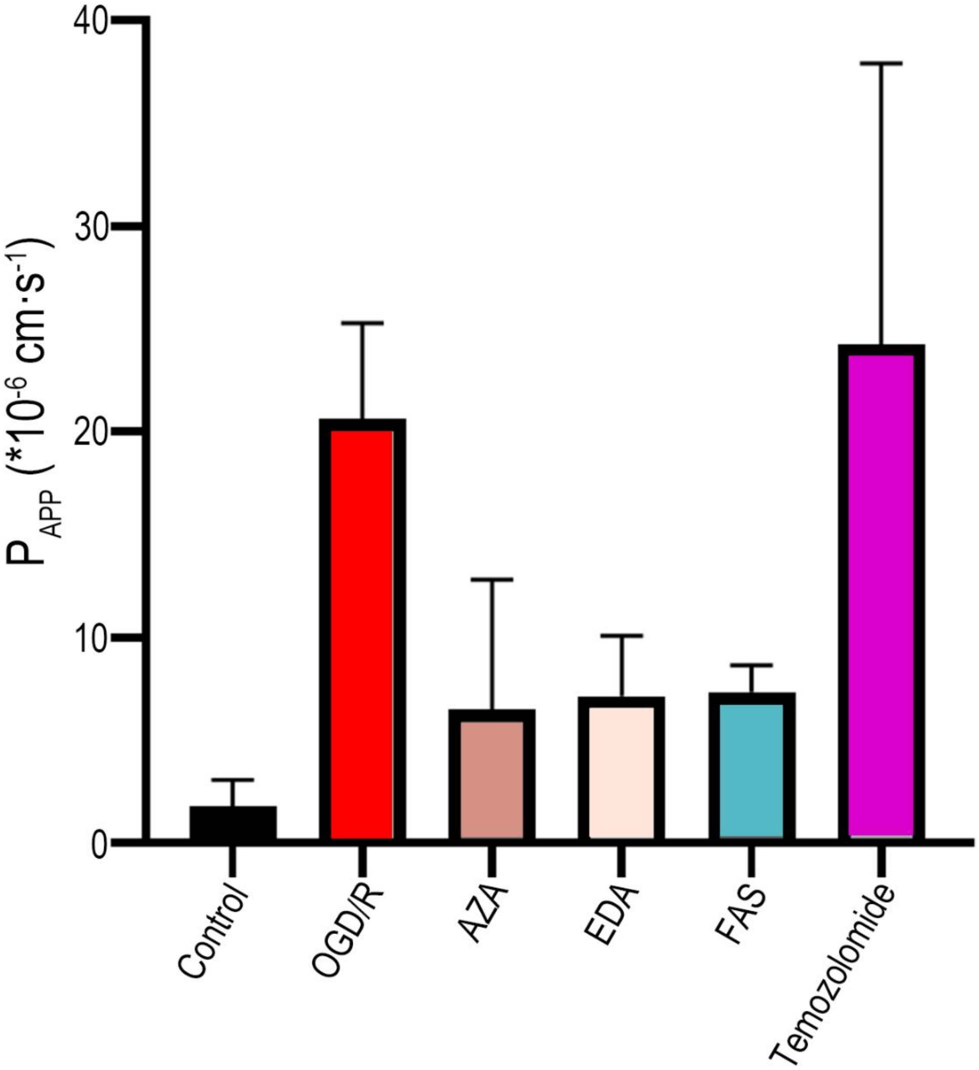

Figure S5. BBB permeability analysis with 4kDa FITC-dextran to preliminary evaluate the effect of AZA, EDA, FAS and temozolomide on OGD/R chips.

Compound	Mechanism of drug action
acetazolamide	A carbonic anhydrase inhibitor which alleviates cerebral edema after ischemic stroke by inhibiting the expression of AQP4.
Edaravone	A free radical scavenger that reduces apoptosis by inhibiting mitochondrial apoptosis pathway.
Fasudil	A Rho kinase inhibitor that maintain BBB integrity by blocking endothelial and pericellular contraction.
temozolomide	An alkylating agent from the imidazotetrazine family.
Glibenclamide	A K_{ATP}/$NC_{Ca\text{-}ATP}$ channel inhibitor.
Imatinib	A tyrosine kinase inhibitor which is helpful to maintain the integrity of BBB.
Citicoline	An intermediate compound that converts choline to phosphatidylcholine that reduces cell membrane rupture.
Prednisolone	A glucocorticoid that has anti-inflammation effect.
Abrocitinib	A JAK1 inhibitor which could improve neuroinflammation and play a neuroprotective role.
Ruxolitinib	An inhibitor of the Janus kinase 1 and 2 that inhibits ferroptosis and reduces neurodegeneration.

Table S1. Summary of drug compounds and mechanisms of drug action.
